# Supplementary material for: Improvement of Mucosal Lesion Diagnosis with Machine Learning Based on Medical and Semiological Data: An Observational Study
Source: J Clin Med. 2022 Nov 7;11(21):6596. doi: 10.3390/jcm11216596 (PMC9654969; doi:10.3390/jcm11216596)
Supplement: Supplementary file 1 [file jcm-11-06596-s001.zip › jcm-1966994-supplementary.pdf]

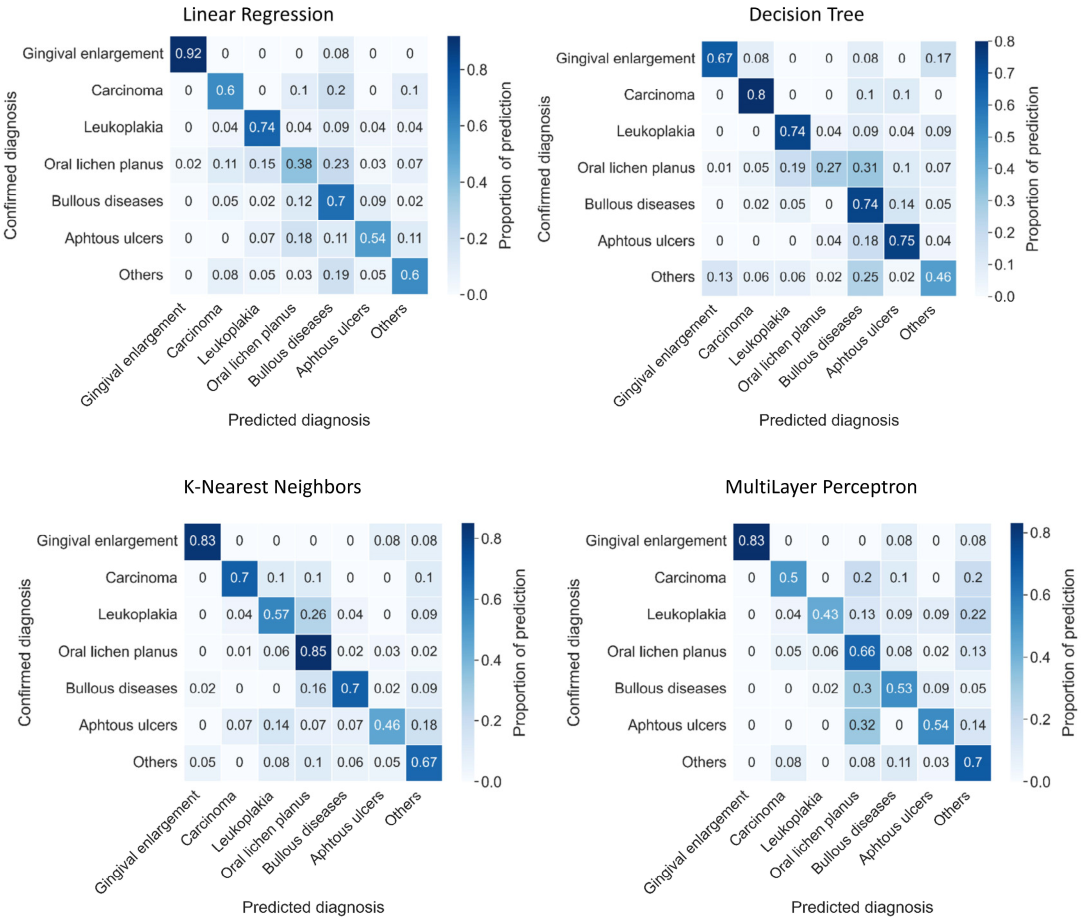

Figure S1: Confusion matrices for the different machine learning models tested on the test dataset. The confusion matrix describes the performance of the difference classifier models on the test dataset.

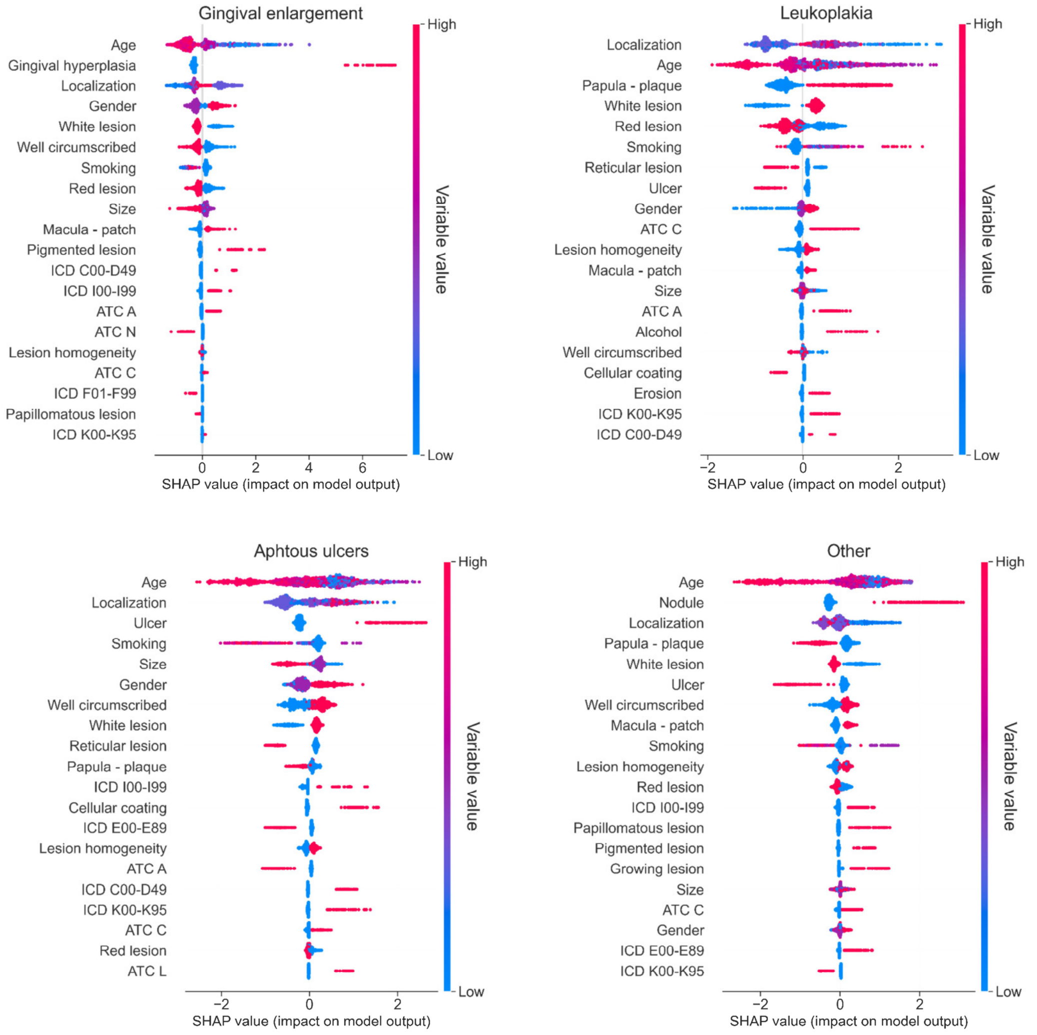

Figure S2: Feature importance for each diagnostic prediction. Each point on the summary plot is a SHAP value for a feature and an instance. The position on the y-axis is determined by the feature and on the x-axis by the SHAP value. The color represents the value of the feature from low to high. Overlapping points are jittered in y-axis direction, so we get a sense of the distribution of the SHAP values per feature. The features are ordered according to their importance.
